# Are we getting the full picture? Animal responses to camera traps and implications for predator studies

**DOI:** 10.1002/ece3.2111

**Published:** 2016-04-06

**Authors:** Paul Meek, Guy Ballard, Peter Fleming, Greg Falzon

**Affiliations:** ^1^ Vertebrate Pest Research UnitNSW Department of Primary IndustriesPO Box 350Coffs HarbourNSW2450Australia; ^2^ School of Environmental and Rural SciencesUniversity of New EnglandArmidaleNSW2351Australia; ^3^ Vertebrate Pest Research UnitNSW Dept Primary Industriesc/‐University of New EnglandArmidaleNSW2351Australia; ^4^ Vertebrate Pest Research UnitNSW Department of Primary Industries1447 Forest RoadOrangeNSW2800Australia; ^5^ School of Science and TechnologyUniversity of New EnglandArmidaleNSW2351Australia

**Keywords:** Behavior, dingo, feral cat, red fox, remote camera, wild dog, wildlife monitoring

## Abstract

Camera trapping is widely used in ecological studies. It is often considered nonintrusive simply because animals are not captured or handled. However, the emission of light and sound from camera traps can be intrusive. We evaluated the daytime and nighttime behavioral responses of four mammalian predators to camera traps in road‐based, passive (no bait) surveys, in order to determine how this might affect ecological investigations. Wild dogs, European red foxes, feral cats, and spotted‐tailed quolls all exhibited behaviors indicating they noticed camera traps. Their recognition of camera traps was more likely when animals were approaching the device than if they were walking away from it. Some individuals of each species retreated from camera traps and some moved toward them, with negative behaviors slightly more common during the daytime. There was no consistent response to camera traps within species; both attraction and repulsion were observed. Camera trapping is clearly an intrusive sampling method for some individuals of some species. This may limit the utility of conclusions about animal behavior obtained from camera trapping. Similarly, it is possible that behavioral responses to camera traps could affect detection probabilities, introducing as yet unmeasured biases into camera trapping abundance surveys. These effects demand consideration when utilizing camera traps in ecological research and will ideally prompt further work to quantify associated biases in detection probabilities.

## Introduction

As the use of camera trapping for ecological research and management continues to grow (McCallum 2013; Rovero et al. 2013; Meek et al. 2015), the limitations of the devices are slowly being elucidated. Advocates for camera traps have often described them as “nonintrusive” (Cutler and Swan 1999; Sollmann et al. 2013; Gregory et al. 2014). We define “intrusive” as ….. *too noticeable … in a way that is disturbing or annoying* (accessed 28 April 2015, http://www.oxforddictionaries.com/definition/learner/intrusive). This is appealing because ethological studies usually require the observations of animals to occur without interference by the observer or measuring devices (Lehner 1996). Changing the behavior of an animal by either attracting them to or repelling them from a sampling device constitutes interference and should be accounted for in analyses of resulting data (Engeman 2005).

In our investigations of mammalian predators in Australia, we have regularly observed indications that animals respond to camera traps. This parallels other researchers' experiences in the Northern Hemisphere; for example, Gibeau and McTavish (2009) reported that camera traps were detected by wolves (*Canis lupus*). Indeed, there are many anecdotal reports suggesting that study animals see and react to camera traps used as survey devices (Séquin et al. 2003; Wegge et al. 2004; Larrucea et al. 2007; Schipper 2007; Gibeau and McTavish 2009; Newbold and King 2009).

Camera traps emit sound and light that can be detected by wildlife, including mammalian predators (Meek et al. 2014a). This may partially explain why predators such as coyotes (*Canis latrans*) behave aversively around camera traps (Larrucea et al. 2007) with some alpha males never conditioning to their presence. It is worth noting that not all reactions to camera traps have been negative; Kelly et al. (2012) suggest some large felids are attracted to camera trap flashes. Smaller predators too, such as stoats (*Mustela erminea*) and feral cats (*Felis catus*), have also been observed displaying a range of behavioral responses to both white flash and infrared camera traps (Bengsen et al. 2011; Glen et al. 2013). Despite these observations, and warnings from authors that behavioral responses to camera traps may affect interpretation of results (e.g., Gibeau and McTavish 2009), few studies have attempted to quantify the responses of animals to camera traps. Gregory et al. (2014) reported no avoidance behavior from four of five arboreal mammal species, nor any from birds and reptiles.

Behavioral responses by animals to camera traps potentially introduce biases to ethological and population ecology investigations (Meek et al. 2014a, 2015) and can violate the assumptions of the survey method by modifying animal behavior (Larrucea et al. 2007); Dénes et al. 2015). Dixon et al. (2009) reported that 73% of red fox (*Vulpes vulpes*) displayed an aversive response in 73% of detections and suggested that this affected their overall detection rate. Further, Wegge et al. (2004) found that camera traps reduced detections for some tigers (*Panthera tigres*) by 50%, thereby impacting upon population estimation.

The issue of fauna responding to camera traps raises several questions: “what stimuli are prompting them?”, “what are the responses?”, “can the camera trap placement be modified to minimize this behavioral response?”, and “when is a response *problematic* for analysis and interpretation?”

Responses of wildlife to camera traps, both positive and negative, must be quantified so that researchers and managers can understand, and where necessary to account for, the influence their investigations are having on fauna. In this study, we sought to evaluate and quantify the range of response behaviors to camera traps displayed by four Australian terrestrial predators during trail‐based surveys. In doing so, we intended to determine whether any of these behaviors were sufficient to impact negatively upon survey outcomes or associated ethological investigations.

## Methods

### Study sites

**Figure 1:**
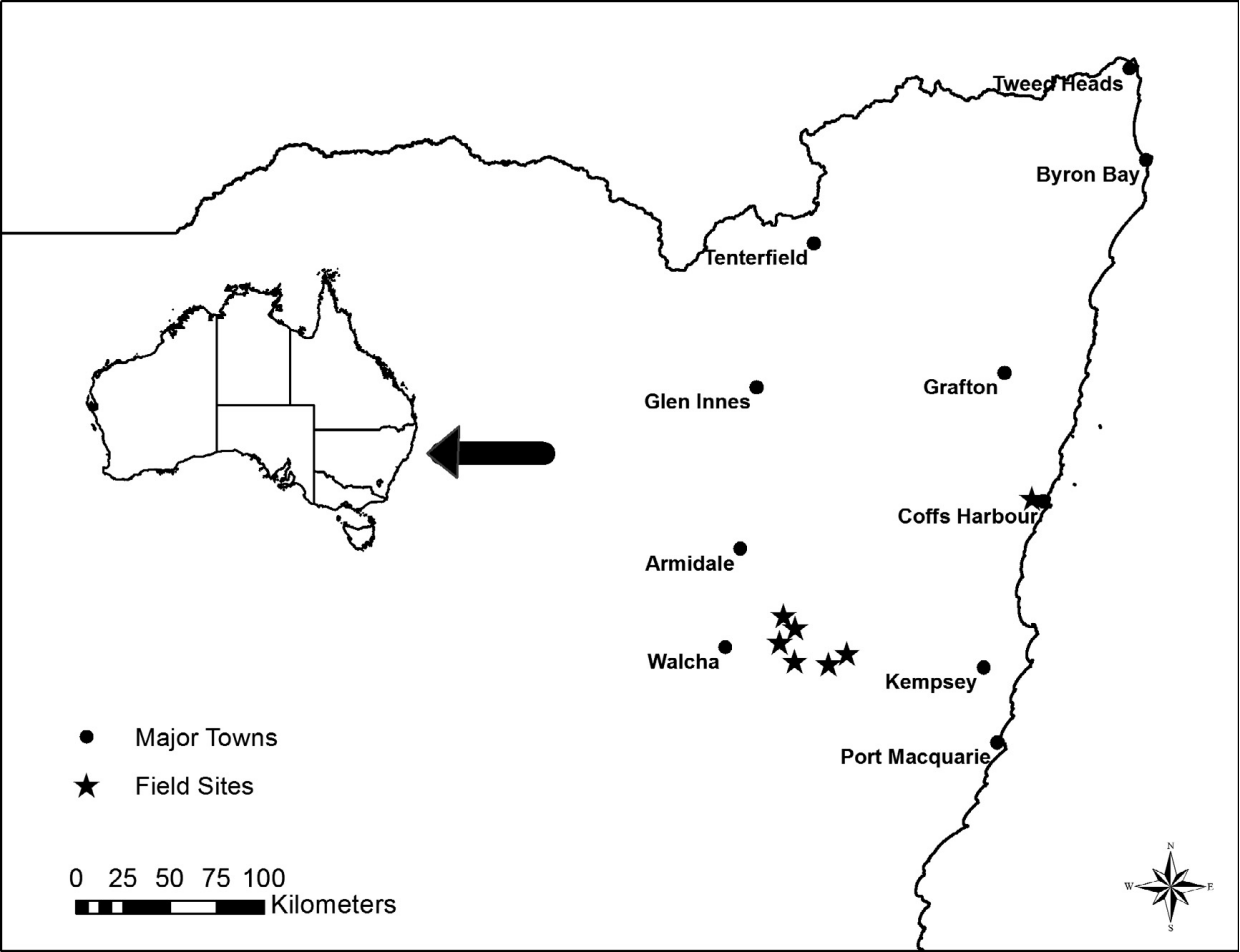
The eight study sites where camera traps were deployed to measure behavioral responses of four predators in NSW, Australia.

Between 2011 and 2014, we conducted surveys at eight sites in northern NSW (Fig. 1. and Table 1). We utilized sites where continuous trail networks permitted camera trap transects of 10–25 km in length. Seven of the sites were located in or near Oxley Wild Rivers National Park (OWRNP; 30°54′15.14″S, 152°07′12.7″E), which is approximately 50 km east of Walcha, NSW, Australia. Oxley Wild Rivers environs are part of the Apsley‐Macleay gorge system and comprise a diverse dry sclerophyll eucalypt forest with grassy woodlands and small patches of mesic forests over metamorphosed and volcanic sediments. Redhill is 8 km west of Coffs Harbour and is a small transect of 5 camera traps (Fig. 1). The site runs along the Great Dividing Range and is dominated by dry sclerophyll eucalypt forests with mesic and subtropical rainforest gullies (30°16′24.77″S, 153°4′7.60″E). The Guy Fawkes site is located in the Guy Fawkes River National Park (29°55′40″S, 152°14′E) and adjacent state forests and has similar vegetation and soils to OWRNP.

**Table 1:** Settings placement details and image records for Reconyx HC600 camera traps used to detect four Australian predators in eight northern New South Wales sites

Site	Year	Spacing (m)	Photos/trigger	Camera traps	Images *n*
Green Gully	2011	1000	5	28	1945
Kunderang Fire Trail	2011	1000	5	36	3133
Moona	2011	1000	5	44	2099
Narrow Neck	2011	1000	5	33	663
Rowleys Creek	2011	1000	5	13	301
Table Top	2011	1000	5	44	700
Guy Fawkes	2012	1000	5	28	1100
Redhill	2014	100–1000	5	5	1505

We deployed 233 Reconyx HC600 camera traps across the sites (Table 1). Camera traps were placed close to the roadside edge, between 50 and 90 cm above the ground and facing along the trail.

### Image processing

From the total dataset of images (*N* = 87,310), we extracted a series of images (*N* = 11,446) of wild dogs (*Canis familiaris)* (Jackson and Groves 2015), foxes, feral cats, and spotted‐tailed quolls (*Dasyurus maculatus*). An event was defined according to Meek et al. (2014b). A temporal buffer of 5 min was used to distinguish between consecutive events within species at a camera trapping station. We subsequently excluded events where it was impossible to clearly observe animal responses, for example, because its face was not photographed or it was not photographed until it had mostly passed the camera trap. For events containing multiple animals, the responses of each individual were scored separately when they responded differently to one another. We only used camera trap data from the first sampling deployment at each site so that we did not introduce a potential bias to the behavioral responses of animals that may have conditioned to the presence of camera traps over time.

There were occasions when an animal's behavior changed as it approached close to the camera trap without it looking directly at the device. Then, it was difficult to ascertain whether the animal's response was to the camera trap or a coincident environmental stimulus and so, taking a conservative approach, we ignored these incidents in our analyses.

We used Renamer^™^ to record the year, site, and location within the filename of each image. EXIFPRO^™^ was used to assign metadata tags for species and behavior (Table 2) to each image. For consistency, one observer (PM) conducted the entire behavioral coding process.

**Table 2:** Ethograms of the possible quantitative behaviors of wild dogs, foxes, feral cats, and spotted‐tailed quolls in response to camera traps (CT) at each stage and phase of a camera trapping event (a), and their facial, movement, and action behavioral responses to camera traps (b). The numbers in brackets in Table (a) relate to the number of response categories in Table (b) that were available for each phase of an event. Although the actions of predators in response to camera traps were observed and classified, occurrences of many were too few for comparisons, so these were omitted from further analysis

(a) Stage	Components	Phase	Quantitative behaviors	
Pre‐CT encounter	Animal	Preencounter travel	Move toward	Move away
Encounter with CT	Animal + CT	Encounter (triggers CT)	No observed response indicating detect CT	Observed response indicating detect CT
		Initial response	No observed reaction	Observe CT (3)
		Movement relative to CT	Stop	Move (4)
		Response to ongoing stimuli	No observed reaction	Observe CT (3)
		Behavior relative to CT	Move (3)	Act (7)
Post‐CT encounter	Animal	Postencounter travel	Move toward	Move away

(b) Observe response	Move response	Act response		
Glance	Startle	Sniff		
Look	Continue	Groom		
	Retreat	Roll		
Stare	Move toward	Mark		
	Move away	Piloerection		
		Sleep		
		Stalk		

### Behavioral assessment

Observing behaviors are complex and open to anthropomorphic interpretation (Lehner 1996). To reduce the potential for observer bias and misinterpretation of behaviors, we devised ethograms to describe the responses of fauna to their interaction with camera traps (Table 2a) and the types of awareness, movements, and actions exhibited by the animals (Table 2b), and scored image sequences (events) corresponding to the quantitative behaviors within them (Table S1). The primary focus of this approach was to quantify the proportion of animal responses to camera traps to determine whether bias was present.

We grouped the quantitative behaviors into responses: Observe, where the animal in the image displayed a visual change to behavior that indicated it had observed the camera trap; startle, where the initial response was a change in facial expression or behaviors, e.g., changing its gait, posture, passage, or velocity of travel, indicating awareness and increased alertness; repulsion, where the animal detected the camera trap and did not continue its passage, and moved away from the camera trap; approach, where the animal detected the camera trap and changes its passage and behavior to move toward or investigate the device; and retreat, a complete flight response. A further class, continue, included all other instances where the animal resumed passage in its original direction after the detection of the camera trap. The specific terms used to code and tag the images have been presented in Table S1.

To determine whether an animal detected a camera when there was no obvious physical response to the camera trap, we also looked for relatively subtle behavioral indicators such as ear twitching (shown by changes in ear position relative to the head in consecutive images), or apparent visual focus on the camera trap. Where these gross or subtle behavioral responses could not be detected, we recorded that the animal did not respond.

Although successive images within a sequence are typically <1 sec apart, it is possible for the gap between sequences to be greater. Consequently, we could not be sure that brief reactions did not occur in this interval between sequences, within an event. As camera traps cannot automatically increase shutter speed, images of animals that changed velocity, following their response to the detection of the device, were often blurred. On occasions, foxes and quolls were moving so fast that only one image was recorded. In these cases, no behavioral response could be attributed.

### Analyses

We assumed independence between events and analyzed behavioral responses as proportions of the population sample with confidence intervals (95%) using a likelihood ratio test (one‐sided confidence interval) in the R program “binom” (Dorai‐Raj 2014) to assess the goodness of fit. We also tested whether predators detected camera traps differentially between day and night, and whether the relative frequency of behaviors differed between night and day.

Results are presented as percentage values for ease of interpretation with 95% confidence intervals for statistical interpretation. A significant difference occurred when the confidence intervals between a set of values did not overlap.

## Results

**Figure 2:**
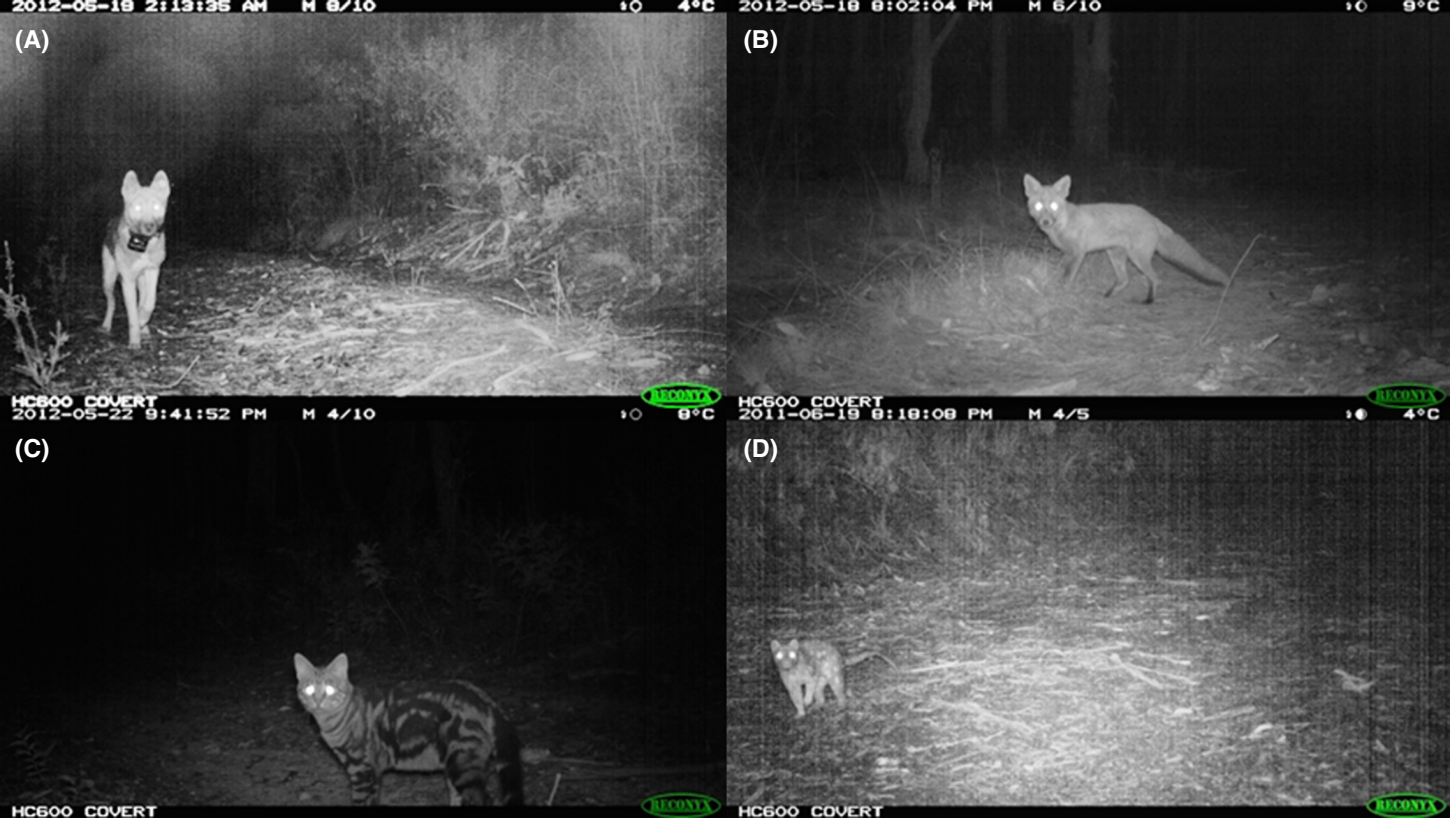
Example nighttime responses of four predators to camera traps with infrared flash. (A) wild dog (with a GPS telemetry collar and an ear tag) looking at and approaching the camera trap, (B) fox displaying a startle response, (C) feral cat staring at the camera trap, and D) spotted‐tailed quoll looking at and approaching a camera trap.

**Figure 3:**
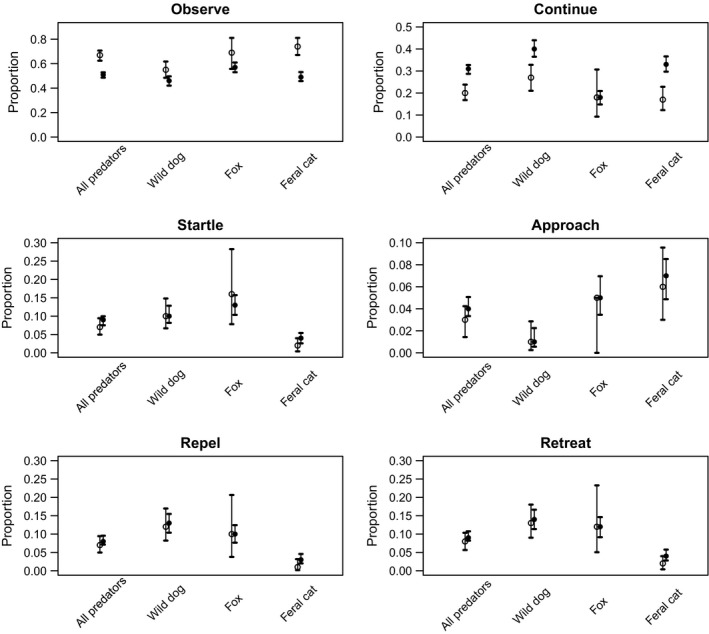
A comparison of six day‐and‐night behavioral responses of four Australian predators to camera traps. Values are proportions to 2 d.p., with 95% confidence interval bars. Open symbols are daytime records, and filled symbols are nighttime records. NB scales are different for each response.

Most images of predators were captured at night (Table 3, e.g., Fig. 2). Quoll records (*N* = 99 events) were substantially fewer than for the other three species so we analyzed them separately. The majority (Fig. 3) of individuals of the three introduced species in camera trap images observed and responded to camera traps.

**Table 3:** Diurnal and nocturnal camera trapping events for four Australian predators during trail‐based surveys

Infrared	Day events	Night events	Total events
All predators	501	2026	2527
Wild dog	214	657	871
Fox	49	598	647
Feral cat	193	704	897
Quoll	–	–	99
Total	957	3985	4983

There was a difference in detection events between day‐and‐night observations when wild dogs, foxes, and feral cats were pooled (Fig. 3), with significantly more animals noticing camera traps during daylight hours. Feral cats detected camera traps more often during the day than at night, while detection of camera traps by both foxes and wild dogs was similar between day and night.

Of the total diurnal events, 49% of predators were moving away from the camera trap when detected (56% of feral cats, 46% of wild dogs, and 39% of foxes), whereas 46% were walking toward the device. Of these predators, 50% of dogs, 45% of foxes, and 40% of feral cats were detected walking toward the camera traps. At night, 51% of predators were detected walking toward (wild dogs 53%, foxes 51%, feral cats 49%), and 43% were walking away (feral cats 48%, wild dogs 41%, foxes 40%) from the camera traps. On 5% of occasions, predators either appeared in front of the camera trap without a determinable entry direction or came from the side.

**Figure 4:**
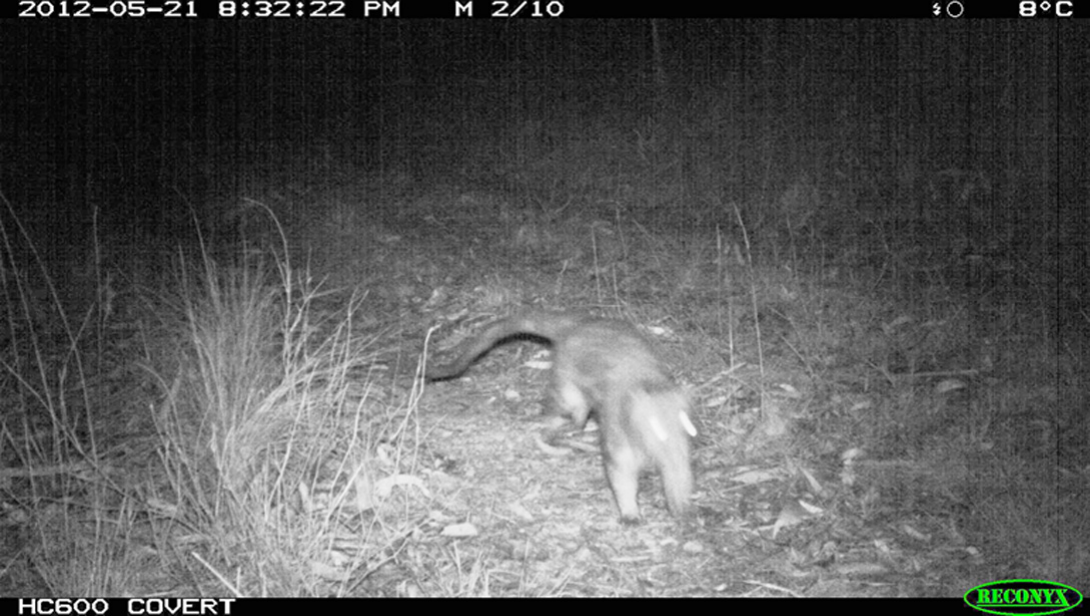
Spotted‐tailed quolls (*Dasyurus maculatus*) often moved too fast on trails to accurately determine their behavioral responses to the camera traps.

Most images of quolls were blurred because they often ran past the camera trap, hindering precise allocation and analysis of behaviors (e.g., Fig. 4). However, we detected most quolls moving toward (57%) or away from camera traps and only few events (2%) where an animal appeared from the side. In most (81%) quoll events, the animal did not show evidence of detecting the camera trap. In 26% of events, the quolls appeared to discover the camera trap and responded negatively (turning away from the camera trap and walking or running back along the track), but only 7% exhibited a startled response.

We found that all predators combined detected camera traps two‐thirds of the time in daytime images (67%; 95% CI = 62.46, 70.7; *n* = 501 events: Fig. 3 Observe) and just over half the time at night (51%; 95% CI = 48.56, 52.91; *n* = 2026 events: Fig. 3 Observe). During the day, feral cats were more likely to notice a camera trap than wild dogs, with foxes overlapping both the other species (Fig. 3 Observe). At nighttime when most images were taken, wild dogs observed the camera traps on less than half of the images and were less likely to observe them than foxes, with feral cats overlapping both other species (Fig. 3 Observe).

Some predators passed the camera traps without showing any behavioral response after discovering the camera trap, although there was a difference between day and night (Fig. 3 Continue). In daylight hours, 20% of predators did not appreciably alter their behavior in response to camera traps and continued past the device (Fig. 3 Continue), whereas 31% did at night. In 8% of daytime events of predators pooled, the animal reversed its direction of travel without passing the device (wild dog 13%, fox 12%, feral cats 2%). Wild dogs and feral cats exhibited different continuation between day and night, with both more likely to continue unaffected at nighttime. Fox continue responses were similar for day and night, although the daytime response showed greater individual variation (Fig. 3 Continue).

There was no difference in the startle responses of foxes, wild dogs, and feral cats in the day or night (Fig. 3 Startle). However, foxes were relatively more likely to display startle behavior at night, and feral cats rarely displayed this behavior irrespective of time of day (Fig. 3 Startle). Similarly, 8% of events of all predators at any time of day resulted in animals being repelled by the camera traps; wild dogs displayed this behavior (12% day, 13% night) more often than foxes (1% day, 1% night), and feral cats were rarely startled by camera traps (Fig. 3 Startle).

Approaches to camera traps after detection were few (maximum 7% by feral cats at night, Fig. 3), with wild dogs at any time of day less likely than nocturnal feral cats and foxes. Foxes and feral cats showed greater variability in approach response during the day.

Wild dogs exhibited similar repel and retreat responses, and these were similar throughout the day (Fig. 3 Repel, Retreat). Likewise, foxes showed similar day‐and‐night repel or retreat responses but with greater individual variation than wild dogs (Fig. 3 Repel, Retreat). Conversely, feral cats were unlikely to be repelled or retreated (<4% of events, Fig. 3) with no difference between day‐and‐night images.

### Incidental observations

A number of other behaviors indicating the detection of and response to camera traps by predators were observed in images. Some individual animals, particularly wild dogs, displayed characteristic behaviors in the presence of camera traps. For example, one individually recognizable animal was never observed walking close to a camera trap and only ever recorded when other members of its social group triggered the camera trap; it was always 20–50 m distant from the camera and observed diverging from the trail as its social group approached a camera trap or rejoining the trail as its social group moved away.

Feral cats often approached camera traps, so much so that camera traps might have acted as a lure. At Green Gully, an adult cat and three kittens all approached the trap after first trigger and stood looking at it while it photographed them. Feral cats were also photographed spraying (scent marking) the camera post. At one site, a cat jumped on top of the camera trap and then jumped down in front of it and sprayed several times.

On many occasions, we saw predators display ear movements in response to the camera traps. Dogs were seen to approach the camera without directly looking at the device, pinning their ears back in a submissive posture (Rogers and Kaplan 2003) and diverting their path to walk around the camera trap. On occasion, feral cats were observed stopping in front of the camera trap and looking at the device and twitching their ears as if to focus on a noise.

Other species, for example, horses (*Equus cabalus*), brown hares (*Lepus europaeus*), red‐necked wallabies (*Macropus rufogriseus*), brush‐tailed possums (*Trichosurus vulpecula*), and feral pigs (*Sus scrofa*), also responded to camera traps during our surveys.

## Discussion

Contrary to previous suggestions about the undetectability of camera traps by target species (e.g., Rowcliffe et al. 2008; Hamel et al. 2013; Trolliet et al. 2014), camera traps were intrusive for the four species of mammalian predators that we studied. Our study showed that most individuals of all four species sensed and responded to camera traps during trail‐based surveys and that these responses were variable. While it is unclear which stimuli prompted discovery and response to the devices, animals responded to camera traps in both the day and night. Camera traps comprise engineered electronic components, for example, PIR sensors and infrared flash, which emit sounds and light, and the animals could have been responding to either emitted stimuli or the physical camera trap and its supporting structures.

Wild dogs would sometimes notice the camera trap, stop, look at the unit, and then reverse their direction of travel (Video S1). In the case of foxes, many displayed responses despite not looking at the camera, suggesting they might be detecting noises (sensu Meek et al. 2014a). Feral cats were recorded stopping, looking at the camera trap and moving their ears, as if attempting to focus on a sound, and often walking to the camera trap (Video S2). Quolls passed camera traps at greater speed than the other three species, but still showed evidence of awareness of and reaction to them. At night, the animals likely observed and responded to the illumination (Meek et al. 2014a), but we were unable to determine whether there was an interaction with noises and light emanating from the cameras.

Over the last decade, there have been an increasing number of analytical methods developed to improve the robustness of surveys and behavioral studies derived from image‐based data (Rowcliffe et al. 2008; O'Brien 2011; Ramsey et al. 2015). However, most analytical tools do not account for detection bias caused by behavioral disturbance to animals from camera trap sounds and light emissions. The responses of animals to camera traps will affect analyses that can be employed (Table S2).

Our investigation was focussed on passive (i.e., no bait), trail‐based surveys so we are cautious about extrapolating our results to active camera trap surveys where an animal is attracted and/or subsequently preoccupied by a lure. In our study, there were instances where the animal in the images was focussing on spoor left by a preceding predator and, at the same time, paid little or no attention to the camera trap. It is conceivable that an animal's preoccupation with the olfactory or visual stimulus of the spoor could have functionally camouflaged the camera trap. Scent posts created by the placement of a camera trap (as evidenced in our study by feral cats spraying the camera trap post) should be added to the list of possible causes of responses to camera traps proposed by Meek et al. (2014a). Further evaluation is warranted to test whether lures act as a distraction, causing animals to ignore camera traps, or conversely to see whether lures introduce additional bias.

There was some evidence that predators were sensing the camera trap before the animal moved into frame, that is, just an ear and eye photographed and no following images. Therefore, our observations of the responses to the devices could underrepresent negative behavioral responses, particularly of the recognition and retreat response in foxes. Sometimes, there was no image of an animal seeing the device, just some of a departing animal. This behavior was recorded often in foxes, and there were many events recorded where foxes and quolls were departing without looking toward the camera.

The responses of feral cats to camera traps in day‐and‐night events were indicative of an animal with acute hearing (Peterson et al. 1969; Heffner and Heffner 1985) and vision (Ewer 1998; Gekeler et al. 2006). However, there were many cases where an animal displayed a negative response when they were within 5–10 m of the camera trap and were startled without looking at the camera trap. Such responses were recorded as a startle response, but in some cases, it was impossible to determine whether the audio outputs were the reason for some less dramatic behavior. We accept that we may have failed to record some subtle behaviors that were hearing related and not visually obvious because it is very difficult to determine exactly when behavior starts and stops (Lehner 1996).

The sensing and response by animals to camera traps were highly variable, and there is little certainty that one species or individual will consistently behave the same around camera traps throughout its life exposure to the devices. We observed individually recognizable cats approaching the camera trap one day and then continuing past the device on the next visit. It is therefore possible that some individuals habituate to the camera traps.

The consequences of animals modifying behavior and changing aversion behaviors to camera traps may have further implications for analysis and need to be understood (Table S2). Most assumptions for abundance estimation require that animals and animal movement are equally distributed throughout the sampling site and period (Rowcliffe et al. 2008; Dénes et al. 2015), that all animals have an equal probability of exposure to a camera trap, and that sources of variation in detection are identified (O'Brien 2011). Where an animal is effected by the presence of a camera trap and displays “shyness,” then many of the repeat measure methods used in measuring populations may be confounded (Lettink and Armstrong 2003; Rowcliffe et al. 2008, 2011; O'Brien 2011; Sollmann et al. 2013; Dénes et al. 2015). Rowcliffe et al. (2011) discuss the effect of animal behavior around camera traps and recommend that randomizing placement will avoid such a bias, although this does not resolve bias related to camera‐trap avoidance where the animal is actively deterred from repeated visits through a startle reaction eliciting avoidance from detection.

In the case of the random encounter model (REM), detection probability is considered to be a function of an animal's position relative to the camera trap (Rowcliffe et al. 2011). Where an animal is camera trap shy or camera trap happy, detection probability is compromised and the assumptions of the method cannot be met. Even the study of animal behavior can be compromised if an animal exhibits atypical behavior (Gibeau and McTavish 2009). Attraction of certain species and individuals to our camera traps would confound assumptions of some other population estimators (Table S2) because the device effectively becomes a lure (Foster and Harmsen 2012).

Failure to detect an individual or misidentifying an animal in an abundance study can lead to adverse ecological decision making, and this issue has been emphasized by Dénes et al. (2015). We are alarmed that the potential error in abundance estimators caused by the detection of and responses to camera traps by animals has been largely overlooked in the literature. Despite a significant effort being made to developing sophisticated analytical methods to analyze data generated by camera traps (see O'Connell et al. 2011), few recognize disturbance effects. The exception being Rowcliffe et al. (2008), who referred to the possible assumption violation of the REM if avoidance of camera traps occurs. Martin et al. (2005) were very clear in their advice on zero inflation, “Understanding how zeros arise and what types of zeros occur in ecological data are more than just semantics; failing to model zeros correctly can lead to impaired ecological understanding.” Zeros elicited from camera traps are no different. There are exceptions where studies have actively assessed camera trap aversion and found no statistical effect on animal behavior (e.g., Gregory et al. 2014). However, in our study, responses to camera traps were commonplace and caused responsive alterations to animal's behavior. Future studies need to quantify these changes to animal behavior in terms of detection and the consequences for analysis of image data. In some studies, animal responses to camera traps are not important (see Table S2). However, this is not always the case so innovative technical and statistical solutions will need to be included in any analysis of camera trap data to address detection bias.

The findings of our research extend the concerns raised by O'Brien (2011) in regard to the bias encountered when using camera traps to estimate abundance, density, and relative abundance. The study by Meek et al. (2014a) and this study provide compelling evidence that camera traps (1) emit sound and light and (2) do affect animal behavior, animals of different sizes, different life histories, and biology. Therefore, it is inappropriate to refer to camera traps as nonintrusive and noninvasive or to credit them for providing undisturbed observations. The future value of using camera traps in population surveys will rely on the recognition of detection errors and the refinement of analytical methods to account for this bias.

## Data Accessibility

Data collected and analyzed in this study will be archived in the library of the University of New England, Armidale, NSW, Australia.

## Conflict of Interest

None declared.

## Supporting information


**Table S1.** An ethogram of terms used to describe the range of behavioural responses of Australian predators to the presence of camera traps.

**Table S2.** The possible effects on population abundance estimators of animals that avoid detection of camera traps due to trap‐shyness or startle behaviours or are attracted to camera traps due to trap happiness or approach behaviours.

**Video S1.** Wild dog behaviour at camera traps.

**Video S2**. Feral cat behaviour at camera traps.
