# Supplementary material for: Are we getting the full picture? Animal responses to camera traps and implications for predator studies
Source: Ecol Evol. 2016 Apr 6;6(10):3216–25. doi: 10.1002/ece3.2111 (PMC4829047; doi:10.1002/ece3.2111)
Supplement: Supplementary file 1 — Table S1. An ethogram of terms used to describe the range of behavioural responses of Australian predators to the presence of camera traps. [file ECE3-6-3216-s001.docx]

**Supplementary Table S1**

An ethogram of terms adapted from Rogers and Kaplan (2003) that were used to describe the range of behavioural responses of Australian predators to the presence of camera traps.

| **Behavioural Unit** | **Description** |
| --- | --- |
| Approach |  |
| AWAYCT | Moving away from the camera trap |
| TOWARDCT | Approaching the camera trap |
| SIDE | Appearing from the side of the camera trap |
| UNKNOWN | No clear direction of passage obvious, a function of camera trap detection failure |
| Recognition of CT |  |
| CTDETECTED | Camera trap recognised by animal, obvious by the visual detection and eye contact recorded in the images |
| CTUNDETECTED | No obvious visual response from the animal, usually as a result of moving away from the device |
| NORESPONSE | Animal directly in front of the camera trap and despite illumination it seems to ignore the device. |
| Behaviour to CT |  |
| STARTLED | Broad classification of an alarm response to the camera trap |
| UNSTARTLED | Broad classification of the animals failure to be alarmed by the camera trap |
| 1^st^ Response to CT |  |
| OBSERVED | Animal registers the camera trap present and continues to display further behaviours |
| ATTRACTED | Animal shows a deliberate attraction by approaching the device |
| REPELLED | Animal is frightened by the device and deterred, moving away from the camera trap |
|  |  |
| 2^nd^ Response to CT |  |
| GLANCE | A brief glance at the camera trap is detected through eye reflection and head movement |
| LOOKATCT | Obvious visual recognition of the device through eye reflection and eye movement |
| STAREWALKPAST | Sustained visual recognition of the device as the animal continues to walk past the camera trap |
| STAREWALKTO | Sustained visual recognition of the device as the animal walks towards the camera trap |
| STARESTOP | Sustained visual recognition of the device but the animal stops moving and stands staring for a series of images |
| SITLOOK | Sustained visual recognition of the device but the animal stops moving, sits down and stares for a series of images |
| WALKROUNDCT | A deliberate avoidance where the animal walks wide of the camera trap, often into the vegetation |
| RUNAROUNDCT | A deliberate avoidance and alarm response where the animal canters or runs wide of the camera trap, often into the vegetation |
| WALKTO CT | Obvious attraction to device where the animal approaches the camera but may not maintain eye contact |
| WALKBACK | Direction of travel changes after detection of the device and the animal turns and walks back in the direction of origin |
| WALKPAST | Direction of travel maintained, no eye contact or head movement is detected and the animal passes the camera trap with no change of pace or direction |
| RETREAT | Fear response where the animal runs back in the direction of origin |
| RUNPAST | Direction of travel maintained, no eye contact or head movement is detected and the animal canters or runs past the camera trap with no change of direction |
| SPRAYCT | Deliberate deposition of urine on the camera trap post/camera trap depicted by tail lifting in feral cats and leg cocking in dogs and foxes. |
| TAILLIFT | Dominant or aggressive posture towards the camera trap |
| BARK | Aggressive vocalising behaviour towards the camera trap |
| Unrelated Behaviour |  |
| SNIFF | Focussed olfactory interest in a scent where the animal has either its nose close to the ground and/or vegetation or raised high in the air |
| GROOM | Scratching, combing or licking the pelage |
| ROLL | Scent marking behaviour where the animal rolls on a specific scent point |
| MARK | Scent marking by urine spraying, leg cocking, defecating or facial gland rubbing |
| PILOERECT | Dominant and aggressive posture where the animals back is arched and the hair on the torso and tail is raised |
| SLEEP | Series of images where the animal has entered a period of inactivity adopting a sleeping posture |
| STALKING | Deliberate and careful gait focussed on a visual or audible stimuli causing the animal to enter a pre-pursuit posture |
| PREY | Visual detection of a prey item in the mouth of the animal |
