# Supplementary material for: Are we getting the full picture? Animal responses to camera traps and implications for predator studies
Source: Ecol Evol. 2016 Apr 6;6(10):3216–25. doi: 10.1002/ece3.2111 (PMC4829047; doi:10.1002/ece3.2111)
Supplement: Supplementary file 2 — Table S2. The possible effects on population abundance estimators of animals that avoid detection of camera traps due to trap‐shyness or startle behaviours or are attracted to camera traps due to trap happiness or approach behaviours. [file ECE3-6-3216-s002.docx]

**Supplementary Table S1.**

The possible effects on population abundance estimators of animals that avoid detection of camera traps due to trap-shyness or startle behaviours or are attracted to camera traps due to trap happiness or approach behaviours**.**

| **Analysis Method/Estimator** | **Assumptions** | **Consequences of CT Detection for Analysis** | **Statistical Solution** | **Reference** |
| --- | --- | --- | --- | --- |
| SINGLE MEASURES | | | | |
| Inventory/Presence/Absence | animals will be detected at least once | Single detection is appropriate, as such CT aversion is unimportant, unless animals are trap-shy | not applicable |  |
| Naïve Occupancy | Species are present for the duration of sampling session. Detectability and probability of occurrence is the same at all sites. | Imperfect detection confound the assumptions of hierarchical occupancy | none | Guillera-Arroita *et al.* 2014, MacKenzie *et al.* 2006, |
| Hierachical Occupancy | Species are present for the duration of sampling session. Detectability and probability of occurrence is the same at all sites. | Imperfect detection confound the assumptions of naive occupancy | none | Guillera-Arroita *et al.* 2014, MacKenzie *et al*. 2006 |
| Behaviour | that the sampling method will not interfere with normal behaviour | behaviour modification, avoidance | none | Romero-Muñoz *et al.* (2010) |
| Activity (Circadian) | that the sampling method will not interfere with normal behaviour | activity modification, avoidance | none | Romero-Munoz *et al*. (2010) |
| Distance and point sampling | Detection probability on the transect is 100%, animals do not move away from observer before sighting. | Trap shyness and avoidance reduces detection, confounding two assumptions. | Correction factor measurement required, estimate using dual observers/ cameras | Lettink and Armstrong 2003. (Dénes, Silveira & Beissinger 2015), Melville *et al.* 2008 |
| REPEAT MEASURES | | | | |
| Photographic Rate | Individually marked animals must encounter camera traps and be recognised, detection probability is optimum | PR is dependent on the number of detections of individuals, trap shyness reduces detection rate and thus confounds assumptions, site-specific detection probability is effected by trap-shyness |  | Carbone et al 2001, Jenelle *et al*. 2002, (Foster & Harmsen 2012) |
| Mark Site Recapture | Individually marked animals must encounter camera traps and be recognised, detection probability is optimum | MSR is dependent on the number of detections of individuals, trap shyness reduces detection rate and thus confounds assumptions, site-specific detection probability is effected by trap-shyness |  | Carbone *et al*. 2001, Foster and Harmsen 2012 |
| Capture Recapture | Individually marked animals must encounter camera traps and be recognised, detection probability is optimum | CR is dependent on the number of detections of individuals, trap shyness reduces detection rate and thus confounds assumptions, site-specific detection probability is effected by trap-shyness |  | Foster and Harmsen 2012, (Chandler & Royle 2013) |
| Spatial Presence-absence model | Animals will move about their home range centres according to some probability distribution | Where CT’s are placed and some animals avoid them, their distribution is biased and the assumption of movements around the HR occur with bivariate normal probability is confounded. |  | (Ramsey, Caley & Robley 2015) |
| Occupancy | Species are present for the duration of sampling session. Detectability and probability of occurrence is the same at all sites. | Imperfect detection violates the assumptions of hierarchical occupancy, albeit less so with higher samples and sites | Large sample sizes | Guillera-Arroita et al 2014, MacKenzie et al 2006 |
| Naïve Occupancy | Species are present for the duration of sampling session. Detectability and probability of occurrence is the same at all sites. | Imperfect detection violates the assumptions of naive occupancy | More complex occupancy models | Guillera-Arroita *et al* 2014., MacKenzie *et al*. 2006 |
| Behaviour mensuration | Sampling method does not interfere with normal behaviour, that animals will repeatedly visit the sampling point | behaviour modification, reduced or repeated visits or changed behaviour | none | (Altmann 1974; Martin & Bateson 1993) |
| Activity (Circadian) | that the sampling method will not interfere with normal behaviour, that animals will repeatedly visit the sampling point | behaviour modification, reduced repeat visits or changed behaviour | none |  |
| Activity Index | that animals will repeatedly visit the sampling point | inconsistent repeat detection confounds the assumptions of index methods | none | Engeman 2005; Sollman *et al*. 2013 |
| Random Encounter Model | that animals move independent of camera trap location, detection probability is a function of animal position relative to camera trap | If CT shyness occurs then independent movement in relation to the camera trap is confounded and detection probability will be diminished by trap shyness of detection further away from CT so as not to be identified |  | Rowcliffe *et al*. 2005, Rowcliffe *et al*. 2011 |
| Generalised linear model (GLM) | Independence in observations | Responses to CT’s vary between species and individuals, avoidance of CT’s by part of the populations confounds the assumption of equal detection |  | (Dénes, Silveira & Beissinger 2015), |
| *N*-mixture models | Animal detections at a site and time have the same detection probability | Responses to CT’s vary between species and individuals, avoidance of CT’s by part of the populations confounds the assumption of equal detection | Use single-visit-N-mixture model? | (Dénes, Silveira & Beissinger 2015) |
